# Supplementary material for: Regulation of rice root development by a retrotransposon acting as a microRNA sponge
Source: eLife. 2017 Aug 26;6:e30038. doi: 10.7554/eLife.30038 (PMC5599236; doi:10.7554/eLife.30038)
Supplement: Supplementary file 1. — TE-gene pairs with potential miRNA competition were selected based on the following criteria: (1) TEs with significant expression levels (RPKM >1); (2) Sequence matching in sense orientation; (3) miRNA-binding sites within the matching regions; (4) Correlated expression patterns. [file elife-30038-supp1.docx]

Supplementary file 1. List of putative TE target mimics in rice. TE-gene pairs with potential miRNA competition were selected based on the following criteria: 1) TEs with significant expression levels (RPKM >1); 2) Sequence matching in sense orientation; 3) miRNA-binding sites within the matching regions; 4) Correlated expression patterns.

| TE | Shared miRNA | Gene | Correlation coefficient |
| --- | --- | --- | --- |
| LOC_Os03g13320 | miR156 | LOC_Os11g05030 | 0.881293928 |
| LOC_Os02g28420 | miR159 | LOC_Os06g17910 | 0.964085026 |
| LOC_Os03g36870 | miR160 | LOC_Os11g36210 | 0.972516535 |
| LOC_Os01g72049 | miR164 | LOC_Os08g19600 | 0.768768229 |
| LOC_Os06g39460 | miR166 | LOC_Os09g12060 | 0.92806048 |
| LOC_Os02g08340 | miR167 | LOC_Os09g12615 | 0.845645376 |
| LOC_Os07g04360 | miR169 | LOC_Os02g47480 | 0.812493355 |
| LOC_Os05g31340 | miR172 | LOC_Os06g04100 | 0.920895269 |
| LOC_Os01g03180 | miR393 | LOC_Os01g03170 | 0.985443194 |
| LOC_Os06g38040 | miR394 | LOC_Os11g46050 | 0.908376556 |
| LOC_Os12g26850 | miR395 | LOC_Os01g08890 | 0.993561683 |
| LOC_Os01g01540 | miR396 | LOC_Os01g63750 | 0.907252573 |
| LOC_Os11g35970 | miR397 | LOC_Os05g03400 | 0.944620267 |
| LOC_Os01g01540 | miR444 | LOC_Os12g12830 | 1 |
| LOC_Os10g01880 | miR528 | LOC_Os01g31310 | 0.85553661 |
| LOC_Os03g35970 | miR529 | LOC_Os10g21396 | 0.978543297 |
