# Supplementary material for: Regulation of rice root development by a retrotransposon acting as a microRNA sponge
Source: eLife. 2017 Aug 26;6:e30038. doi: 10.7554/eLife.30038 (PMC5599236; doi:10.7554/eLife.30038)
Supplement: Supplementary file 2. [file elife-30038-supp2.docx]

Supplementary file 2. Sequences of primers used in this study.

| Primer | Sequence (5’ → 3’) |
| --- | --- |
| OsSCL8_RT-F | TAAGGTTGTCGTGGCTATTG |
| OsSCL8_RT-R | ACGCTCCTCCATGCTATAGC |
| OsSCL21_RT-F | CTATTGACTATGGCAGTGATC |
| OsSCL21_RT-R | TGCTGATGTTAGCACTGTCCG |
| OsSCL21_2D_31320_genotype-F | TCTTCCTCCTCCACAACCAG |
| OsSCL21_2D_31320_genotype-R | TTACTGACAGCATCCGCATC |
| OsSCL21_2A_00410_genotype-F | AAAACCATCGTCGAGCAAAG |
| OsSCL21_2A_00410_genotype-R | GATATGGAGGGAAGGGGATC |
| OsSCL29_RT-F | GTGTCGTGAAGGAGCTCCGC |
| OsSCL29_RT-R | CGCCAACATGGACTGCAACG |
| AtSCL6_RT-F | CGATTTCGACGTTGGATATG |
| AtSCL6_RT-R | AGAAGGTGGAGGAGCAAAGA |
| MIKKI_spliced_RT-F | CATATTGTCAGGGTTCAATC |
| MIKKI_unspliced_RT-F | ACGTTGACACATATCATACC |
| MIKKI_RT-R | TCTTGGAACCATCAGTGAAC |
| eEF1α_RT-F | GCACGCTCTTCTTGCTTTCACTCT |
| eEF1α_RT-R | AAAGGTCACCACCATACCAGGCTT |
| UBQ10-F | GGCCTTGTATAATCCCTGATGAATAAG |
| UBQ10-R | AAAGAGATAACAGGAACGGAAACATAGT |
| pri-ath-miR171-F | TTGATATTGGCCTGGTTCAC |
| pri-ath-miR171-R | TATTGGCGCGGCTCAATCAG |
| pri-osa-miR171b-F | GGGAGAGTGCGATGTTGG |
| pri-osa-miR171b-R | GCAAGCTTGTGATATTGG |
| pri-osa-miR171c-F | GTGGGAACGGGATATTGG |
| pri-osa-miR171c-R | AAGGCGACGTGATATTGG |
| pri-osa-miR171d-F | TTGTAGCTATGATGTTGG |
| pri-osa-miR171d-R | ATGGTGCTGAGATATTGG |
| pri-osa-miR171e-F | TGGTAGCTATGATGTTGG |
| pri-osa-miR171e-R | AGAGCACTAAGATATTGG |
| pri-osa-miR171f-F | GGGAGAGTGCGATGTTGG |
| pri-osa-miR171f-R | GCAAGCTTGTGATATTGG |
| pri-bdi-miR171-F | CCAAGGATTACTATACTGGC |
| pri-bdi-miR171-R | CGGTACAGGTTGCTAGGCAA |
| osa-miR171b_northern | GATATTGGCACGGCTCAATCA |
| U6_northern | TATGCGTGTCATCCTTGCGCAG |
| MIKKI_ChIP-F | GAACACATGAAGAAGTATGC |
| MIKKI_ChIP-R | TCCTCAATACTTACCTTGTC |
| MIKKI_BS-F | AAGAAAAAGAAAGGTAATAGGAGAT |
| MIKKI_BS-R | TAATCCTRCCCTCCATCCTATATAA |
| MIKKI_CRISPR gRNA-F | GGCAACCCTGACAATATGGCCTTT |
| MIKKI_CRISPR gRNA-R | AAACAAAGGCCATATTGTCAGGGT |
| MIKKI_genotype-F | GGTTTGATGTGGCCAAATGTG |
| MIKKI_genotype-R | CTTCCTCCAACTCAAAGTCAG |
