# Supplementary material for: Regulation of rice root development by a retrotransposon acting as a microRNA sponge
Source: eLife. 2017 Aug 26;6:e30038. doi: 10.7554/eLife.30038 (PMC5599236; doi:10.7554/eLife.30038)
Supplement: Supplementary file 3. [file elife-30038-supp3.docx]

Supplementary file 3. Tissues selected for Arabidopsis TE coexpression analysis.

| Samples | SRR IDs |
| --- | --- |
| anther | SRR3581684 |
| carpel | SRR3581851 |
| dry seed | SRR3581731 |
| flower at stage 1 | SRR3581859 |
| flower at stage 9 | SRR3581865 |
| flower at stage 19 | SRR3581702 |
| germinating seeds at stage 2 | SRR3581733 |
| leaf | SRR3581847 |
| petal | SRR3581688 |
| root | SRR3581356 |
| developing seeds at stage 7 | SRR3581881 |
| sepal | SRR3581855 |
